# What Is the Tree of Life?

**DOI:** 10.1371/journal.pgen.1005912

**Published:** 2016-04-14

**Authors:** W. Ford Doolittle, Tyler D. P. Brunet

**Affiliations:** Department of Biochemistry and Molecular Biology, Dalhousie University, Halifax, Nova Scotia, Canada; Baylor College of Medicine, UNITED STATES

## Abstract

A universal Tree of Life (TOL) has long been a goal of molecular phylogeneticists, but reticulation at the level of genes and possibly at the levels of cells and species renders any simple interpretation of such a TOL, especially as applied to prokaryotes, problematic.

One of the several ways in which microbiology puts the neo-Darwinian synthesis in jeopardy is by the threatening to “uproot the Tree of Life (TOL)” [1]. Lateral gene transfer (LGT) is much more frequent than most biologists would have imagined up until about 20 years ago, so phylogenetic trees based on sequences of different prokaryotic genes are often different. How to tease out from such conflicting data something that might correspond to a single, universal Tree of Life becomes problematic. Moreover, since many important evolutionary transitions involve lineage fusions at one level or another, the aptness of a tree (a pattern of successive bifurcations) as a summary of life’s history is uncertain [2–4].

## Darwin’s Tree of Life Hypothesis

Before 1859, hierarchical (tree-like) patterns of organismal relationships were commonly drawn and taken to mirror some natural order, most likely divine [5]. What Darwin gave us when he published *The Origin of Species* was a nontheistic reason for the discoverability and utility of such classifications. “Community of descent is the hidden bond which naturalists have been unconsciously seeking, and not some unknown plan of creation,” he wrote [6]. There was to be an actual TOL whose “ramifying branches may well represent the classification of all extinct and living species in groups subordinate to groups” [6]. Bapteste and one of us have called this claim by Darwin his “TOL Hypothesis”—that is, that the tree-like pattern of relationships recognized by systematists reflects an underlying tree-like evolutionary process [7].

After Darwin, classifications were most often assumed without proof to be evolutionary: phenetics was taken to be identical to phylogenetics. As De Querioz noted, “…the relationships expressed in existing taxonomies were merely reinterpreted as the result of evolution, and evolutionary concepts were developed to justify existing methods” [8]. Not much could be done to improve on this as long as the methods of comparative biology (mostly anatomical) remained the basis for classification, as they had been for centuries.

In the mid-1960s, molecular sequencing (first proteins, then ribosomal RNAs, then genes, and now genomes) appeared to offer a way out of such circularity, a possible independent proof of the TOL hypothesis, some thought. Emile Zuckerkandl and Linus Pauling wrote:

“…molecular phylogenetic trees should in principle be definable in terms of molecular information alone. It will be determined to what extent the phylogenetic tree, as derived from molecular data in complete independence of organismal biology, coincides with the tree constructed on the basis of organismal biology…”

And thus,

“…If the two trees are mostly in agreement with respect to the topology of branching, the best available single proof of the reality of macro-evolution would be furnished” [9].

This made a lot of sense at the time. The way in which genes replicate and mutate is, barring recombination, tree-like. One consequence is that ancestral nodes in a tree constructed from gene sequences are interpreted as corresponding to real ancestors (actual genes). Nothing like that would necessarily be the case for trees based on “organismal biology,” that is, on phenotypic similarities and differences. If evolution were not the cause of phenotypic similarities and differences, then phenetic classifications would be like those used to order books in a library: all the books on Canadian cuisine might (arguably) belong on the same shelf, but no one would claim that they descended from one first book on that subject. Nodes in such a phenetic classification need not represent ancestors [7].

So, agreement between trees would indeed have been some sort of “proof” of Darwin’s TOL hypothesis, with two caveats. The first is that no widely accepted prokaryotic tree “constructed on the basis of organismal biology” was actually available for comparison: microbiologists had given up the attempt to make one in the mid-1950s, and no one since has been foolish enough to reboot the effort. The second is that agreement of molecular and organismal trees is not really a disproof of the theistic explanation Darwin wanted to supplant. Any sensible creator would surely use similar genes to make similar organisms.

The molecular phylogenetic tree-making enterprise has grown more spectacularly than Zuckerkandl and Pauling could have dared hope, and, in particular, the use of small-subunit ribosomal RNA (ssu rRNA) as “molecular chronometer” has revolutionized systematics, most importantly making it possible to put all prokaryotes and all eukaryotes into a single universal TOL. The story of how Carl Woese courageously pioneered the use of this molecule has been often told [10,11]. And the discovery by Woese and George Fox that prokaryotes were deeply divided into Bacteria and Archaea, so that life appeared to comprise three domains, not two (Prokaryotes and Eukaryotes), has by now made it into all the textbooks.

That LGT might be a spoiler for the TOL was only a suspicion at the beginning of the era of molecular phylogenetics. Japanese clinical microbiologists found in the early 1960s that antibiotic resistance could spread between bacterial species via “resistance transfer factors,” now called plasmids [12]. But at that time, such cases were thought to be special, disease-related, and possibly human-caused. Sporadic cases of incongruence between protein-coding and rRNA gene trees were subsequently discovered [13,14], but it was probably the evidence from complete bacterial genome sequences that gave rise to the first serious questioning of the TOL. The claim that a quarter of the genes of the thermophilic bacterium *Thermotoga maritima* had been transferred from Archaea [15] stunned many of us when it appeared in 1999, and prompted one of us to write that if…

“…different genes give different trees, and there is no fair way to suppress this disagreement, then a species (or phylum) can ‘belong’ to many genera (or kingdoms) at the same time: There really can be no universal phylogenetic tree of organisms based on such a reduction to genes” [2].

## Nearly Universal Trees and the Statistical Tree of Life

A decade and a half of prokaryotic tree-making has not produced general agreement on just how much LGT there is, other than “much more than we expected,” although there have been many serious attempts [16]. Indeed, there is little agreement on how to quantify LGT as a process and its impact on genomes over all of evolutionary time. The most reasonable way, we think, is to ask of any contemporary genome how many of its genes have been strictly vertically inherited along a lineage of replicating genomes, tracing all the way back to that of the last universal common ancestor (LUCA). There are only about 100 genes that are found in all or nearly all prokaryotic genomes and thus have a strong claim to having been present continuously in all lineages [17,18]. These are largely involved with the ribosome and its functions (or transcription), and are considered to be relatively immune to LGT, according to the “complexity hypothesis” [19].

The complexity hypothesis is the notion that genes whose products interact with the products of many others will have so many coevolved molecular dependencies that function in a distantly related cellular environment is impaired. “Connectivity” (number of protein–protein interactions), not functional category, is in fact the key determinant [20], and there are highly connected systems other than translation in many cells. So it may be equally relevant that translation and transcription are conservative and essential cellular activities, while many frequently transferred genes function in “optional” metabolic systems, representing alternative and therefore evolutionarily more changeable ways cells can make livings [18].

Puigbo et al. [21,22] have for some time been conducting large-scale gene family tree comparisons across bacteria and archaea, the Forest of Life (FOL) project involving thousands of such families. Even among their 102 Nearly Universal Trees (NUTs), made from genes found in all or nearly all bacteria and archaea, there are very few with identical histories. However, NUT topologies are “far more congruent than expected by chance,” so “they appear to reflect a significant central trend, an attractor in the tree space that could be equated with the STOL [the Statistical Tree of Life]” [22].

## Pangenomics

Most gene families are not among the NUTs, however, and even within a designated species, a sizeable fraction of any genome participates in rapid gene loss and gain (by LGT). The pangenome concept, which aims to describe the gene repertoire of a bacterial species by comparing gene contents of several to many of its strains, supports this notion. A typical bacterial genome comprises a “core” of genes present in all or nearly all of the strains of its species and often many more “dispensable” or “accessory” genes [23] present in only some strains (as few as one). With *Escherichia coli* (several thousand genomes sequenced), the average strain carries about 5,000 genes. Genes shared between all or almost all strains number little more than 3,000, but the number of gene families with a representative in at least one *E*. *coli* genome is approaching 100,000 [24,25]. *Prochlorococcus*, the world’s most plentiful organism and most important oxygen-provider, shows an average genome content of only about 2,000 genes but a pangenome calculated at 85,000, so far [26,27]. Archaea, too, boast pangenomes [28], and we have come to think in terms of “distributed” prokaryotic genomes and “shared genomic resource” models, in which genes are lost when superfluous and regained when needed, by LGT.

According to a “strong version” [29] of the popular Black Queen hypothesis [30], different strains (or species) will lose different genes for “leaky” synthetic functions—those whose products leak out of cells and thus allow “cross-feeding.” They become mutually dependent. Several recent metagenomic surveys have uncovered heterogeneous populations of previously unknown small-genomed bacteria or archaea that may be metabolically dependent on each other in this way [31,32]. In line with this, Wolf and Koonin [33] suggest a general model in which gene loss is the dominant mode of prokaryotic evolution: what saves genomes from extinction is periodic genomic expansion, in part via multiple simultaneous (or nearly simultaneous) LGT events. Whether such a punctuational pattern holds or gene gain and loss are more regularly ongoing processes, the result is that often less than half of the genes in any strain’s genome are likely to have been continuously present along a lineage of genomes stretching back to the first member (common ancestor) of that species.

As we go deeper than species into the TOL, the fraction of vertically inherited genes can only get smaller. In an influential 2009 analysis, Lapierre and Gogarten extended and deepened the pangenome analysis to 573 bacterial genomes spanning the then available diversity [34]. The core, about 250 genes, which would have included the NUTs, comprises 8% of the typical bacterial genome and would shrink further if archaea were included. Among dispensable genes, they called 64% “character genes”—“essential for colonization and survival in particular environmental niches” and found in related species—while the 28% more rapidly turning-over genes were strain-specific “accessory genes.”

## Dreams of LUCA

What, then, did LUCA’s genome contain? At one extreme, one might imagine that all contemporary gene families have their coalescents (roots) or direct ancestors thereof, in the genome of LUCA. On this model, either (i) that genome was large, and descendant lineages have differentially lost gene families, or (ii) many important and widely distributed gene families have evolved since LUCA and are not detectably homologous to other gene families with which they share common origins. Mixtures of (i) and (ii) might also be entertained.

At the other extreme, one could hold that LUCA as a cell had a “normal” or even smallish prokaryotic genome (1,500–5,000 genes) with only 100 or so genes (represented in NUTs) that might potentially have been passed down directly to all or almost all contemporary genomes. This would be a “uniformitarian” model, explaining the past in terms of processes familiar from the present. In all descendant lineages, the majority of LUCA’s genes will have been replaced via LGT—from closely related and distant lineages of other genes, homologous or not, carrying out the same or quite different cellular functions (structural and metabolic). A key difference from the first model is that some contemporary gene families will have had coalescents that predate LUCA [35–37]. That is, the donors for many LGT events would have been cellular lineages that have since gone extinct, because LUCA is by this and the previous conceptualization the last universal common cellular (not necessarily genomic) ancestor.

More explicitly, according to the second model, the fraction of any particular contemporary prokaryote’s genes that have been strictly vertically inherited along a lineage of replicating genomes tracing all the way back to LUCA would comprise a universally conserved core (roughly the NUTs) plus “character genes” defining its metabolic lifestyle—methanogenesis genes if both LUCA and the contemporary prokaryote were methanogens, for instance. If the contemporary prokaryote we picked was instead a cyanobacterium while LUCA was methanogenic, non-core shared genes would be substantially fewer. Given that even within the NUTs there is some incongruence of trees, the fraction of a randomly chosen or “average” contemporary prokaryote’s genes that have been inherited only vertically since LUCA could easily be only a small percentage.

A third model, favored by some, is to redefine LUCA as a community, not a single cell or species [38,39]. Although in our view conflating “having common ancestry” with “having a common ancestor,” this model points out the basic conflict implicit in reconstructing the TOL from the sequences of genes that disagree. We are using genes as a proxy. What we have really been after is a tree of entities at some level of the biological hierarchy higher than genes or genomes—cells, organisms, populations, or (most likely) species. Perhaps our quest should be recast as simply an attempt to retrace life’s history at such a higher level, and not as an effort to achieve the proof of the “TOL Hypothesis” that Zuckerkandl and Pauling thought gene sequences could provide. Perhaps we have already unknowingly done this: Tassy recently mused that “we enter a world of non systematic phylogenetics, a surprising oxymoron” [40].

## Trees of Cells and Species

If one assumes, almost as an entailment of Schleiden and Schwann’s cell theory, that all cells derive from previous cells, then it could be the case that there is a single tree-like pattern relating all cells, although one would need to look up to the level of “species” in lineages where sex became a reproductive necessity. Although incongruence of gene trees due to LGT means that no single one can be taken as topologically equivalent to such a tree of cells and species, the STOL might still be the best (though not a guaranteed) proxy for it. Patrick Forterre endorses this common view when he writes:

“The universal tree should depict evolutionary relationships between domains defined according to the translation apparatus reflecting the history of cells (and their envelope) and not according to the global genome composition that is influenced by LGT, virus integration and endosymbiosis, the history of which is incredibly complex” [41].

But we might also ask what warrant there is to believe in such a tree-like history of cells, coupled or not to molecular phylogenies. Reticulating events that would compromise trees at the cellular level are occasional or regular whole-cell fusions, mixing entire genomes and, more contentiously, conjugation and the compromising of cellular integrity represented by phage attachment or transformation. These latter introduce only a little genetic material each time, but when summed over many events, can replace every gene: the argument would be over whether the pattern of material as well as genetic relationships might be seen as non-tree-like.

Interspecies whole-cell fusion resulting in heterodiploid cells does occur in haloarchaea [42] but is probably rare in other prokaryotes. Rare does not mean evolutionarily unimportant, however. Whatever theory we might support as to the origin of eukaryotes, we must acknowledge that something like a fusion of (presumably unicellular) archaeal and bacterial cells was instrumental in this most “basic” divergence. Although a claim that many major archaeal phyla were independently founded by the simultaneous importation of many bacterial genes [43] is now in doubt [44], such events as the acquisition of suites of genes for respiration by the anaerobic methanogenic ancestors of haloarchaea are hard to reconcile as one-gene-at-a-time LGT events. And there is no doubt that a fusion of cyanobacterial and eukaryotic cellular lineages ultimately gave rise to higher plants and, through secondary and tertiary endosymbiotic events, to several algal lineages.

So, even a tree of cellular lineages is not an unproblematic concept. Students of animals and plants have long accepted that incomplete lineage sorting, introgression, and full-species hybridization pose difficulties for the sorts of trees that Darwin might have had us draw. But it is microbes, with their promiscuous willingness to exchange genes between widely separated branches of any “tree,” that have most seriously jeopardized the neo-Darwinian synthesis, in the oversimplified form that we have often presented it to the public [45]. More sophisticated understandings do remain possible [3] and should be debated in a more conceptually and science-historically self-aware context [4].
